# Overexpression of an antimicrobial peptide derived from *C. elegans* using an aggregation-prone protein coexpression system

**DOI:** 10.1186/2191-0855-3-45

**Published:** 2013-08-15

**Authors:** Satoshi Tomisawa, Eri Hojo, Yoshitaka Umetsu, Shinya Ohki, Yusuke Kato, Mitsuhiro Miyazawa, Mineyuki Mizuguchi, Masakatsu Kamiya, Yasuhiro Kumaki, Takashi Kikukawa, Keiichi Kawano, Makoto Demura, Tomoyasu Aizawa

**Affiliations:** 1 Graduate School of Life Science, Hokkaido University, Sapporo, Hokkaido 060-0810, Japan; 2 R&D Center, Nippon Meat Packers, Inc., 3-3 Midorigahara, Tsukuba, Ibaraki 300-2646, Japan; 3 Center for Nano Materials and Technology (CNMT), Japan Advanced Institute of Science and Technology (JAIST), 1-1 Asahidai, Nomi-shi, Ishikawa 923-1292, Japan; 4 National Institute of Agrobiological Sciences, Owashi 1-2, Tsukuba, Ibaraki 305-8634, Japan; 5 Faculty of Pharmaceutical Sciences, University of Toyama, 2630 Sugitani, Toyama 930-0194, Japan

**Keywords:** Antimicrobial peptide, Coexpression, Inclusion bodies, Refolding, NMR, HSQC

## Abstract

Antibacterial factor 2 (ABF-2) is a 67-residue antimicrobial peptide derived from the nematode *Caenorhabditis elegans*. Although it has been reported that ABF-2 exerts *in vitro* microbicidal activity against a range of bacteria and fungi, the structure of ABF-2 has not yet been solved. To enable structural studies of ABF-2 by NMR spectroscopy, a large amount of isotopically labeled ABF-2 is essential. However, the direct expression of ABF-2 in *Escherichia coli* is difficult to achieve due to its instability. Therefore, we applied a coexpression method to the production of ABF-2 in order to enhance the inclusion body formation of ABF-2. The inclusion body formation of ABF-2 was vastly enhanced by coexpression of aggregation-prone proteins (partner proteins). By using this method, we succeeded in obtaining milligram quantities of active, correctly folded ABF-2. In addition, ^15^ N-labeled ABF-2 and a well-dispersed heteronuclear single quantum coherence (HSQC) spectrum were also obtained successfully. Moreover, the effect of the charge of the partner protein on the inclusion body formation of ABF-2 in this method was investigated by using four structurally homologous proteins. We concluded that a partner protein of opposite charge enhanced the formation of an inclusion body of the target peptide efficiently.

## Introduction

Antimicrobial peptides play an important role in innate immunity as a part of the host defense response (Ganz 2003; Radek and Gallo 2007). Antimicrobial peptides are thought to kill bacteria by breaking their cell membranes, although the exact mechanisms are still unclear (Sato and Feix 2006; Mani et al. 2005). To date, numerous antimicrobial peptides have been identified in a wide range of organisms, such as mammals, insects, and plants (Zasloff 2002).

The nematode *Caenorhabditis elegans* has been successfully used as a model species in many fields of biological research (Kaletta and Hengartner 2006; Walhout et al. 2000). Due to its lack of an adaptive immune system, this tiny worm relies solely on its innate immune defense to cope with pathogen attacks. Therefore, the worm is widely used in the study of host innate immunity, and antibacterial molecules related to its innate immunity have been identified (Schulenburg et al. 2004; Roeder et al. 2010).

Antibacterial factor (ABF) is an antimicrobial peptide identified in *C. elegans* (Kato et al. 2002). ABF was first found by a computer-assisted search of a database using the amino acid sequence of *Ascaris suum* antibacterial factor (ASABF) (Zhang et al. 2000). Both ABF and ASABF are thought to belong to a cysteine-stabilized α-helix and β-sheet (CSαβ) superfamily, which contains a single α-helix and a pair of anti-parallel β-sheets stabilized by three or four disulfide bridges. Until now, six kinds of ABF (ABF-1 ~ 6) have been identified from *C. elegans* (Froy 2005). However, the antimicrobial activity has been characterized for only one of these ABFs, ABF-2. A previous study reported that ABF-2 has a broad antimicrobial spectrum compared to that of other CSαβ-type antimicrobial peptides. It has been reported that the C-terminal region of ABFs is longer and more widely diverse than that of other CSαβ-type antimicrobial peptides. Moreover, ABFs differ from the “classical” CSαβ-type antimicrobial peptides, such as drosomycin and plant defensins, in terms of the spacing of half-cystine residues, cysteine pairings, and the organization of the precursor. Therefore, it is thought that the structural properties of ABFs may contribute to their broad antimicrobial spectrum. Although the CSαβ structure of ASABF was solved by ^1^H-NMR (Aizawa et al., manuscript in preparation), the structure of ABF-2 remains unclear. Therefore, the structural analysis of ABF-2 will provide new clues to clarify the structure-activity relationships of ABFs.

In general, the recombinant production of antimicrobial peptides has been difficult because of their activity, although such production has used successfully in a number of studies. In addition, ABF-2 contains four intramolecular disulfide bridges, and thus it is difficult to produce correctly folded and active ABF-2. There are two major methods for producing recombinant peptides that require the formation of disulfide bridges for proper folding and function. One method uses the yeast secretory expression system. Recombinant peptide expression in yeast has many advantages, such as disulfide bridge formation and proper folding (Porro et al. 2005). The yeasts *Pichia pastoris* and *Saccharomyces cerevisiae* are widely used as expression hosts for recombinant peptides. However, a previous study reported that the expression level of ABF-2 in *P. pastoris* was extremely low (100 μg/L) (Kato et al. 2002). In addition, in the case of overexpression of ASABF by using *P. pastoris*, it was reported that unfavorable degradation occurred in the C-terminus (Zhang et al. 2000). The other method is to refold the inclusion body into peptides with native conformations (Singh and Panda 2005). In this study, we chose *E. coli* as an expression host and tried to express ABF-2 as an inclusion body in *E. coli.* Unfortunately, when ABF-2 was directly overexpressed, its expression as an inclusion body in *E. coli* was low. In this study, therefore, we applied a coexpression method for the production of ABF-2 to enhance the inclusion body formation. In this method, the coexpression of an aggregation-prone protein (partner protein) was expected to enhance the inclusion body formation of the peptide of interest (target peptide), and to protect the target peptide from proteolytic degradation by protease. Moreover, we evaluated the effect of the charge of partner proteins on the inclusion body formation of ABF-2 in this method by using four structurally homologous proteins. As far as we know, this is the first report to show experimentally that the isoelectric point of the coexpressed partner protein is an important factor for inclusion body formation of the target peptide.

## Materials and methods

### Materials

*E. coli* DH5α was used as a host strain for cloning and for preparing template plasmids. *E. coli* BL21(DE3) was used as an expression host. ^15^ N-labeled CHL medium was purchased from Chlorella Industry.

### Construction of a vector coexpressing ABF-2 and a partner protein

**Figure 1:**
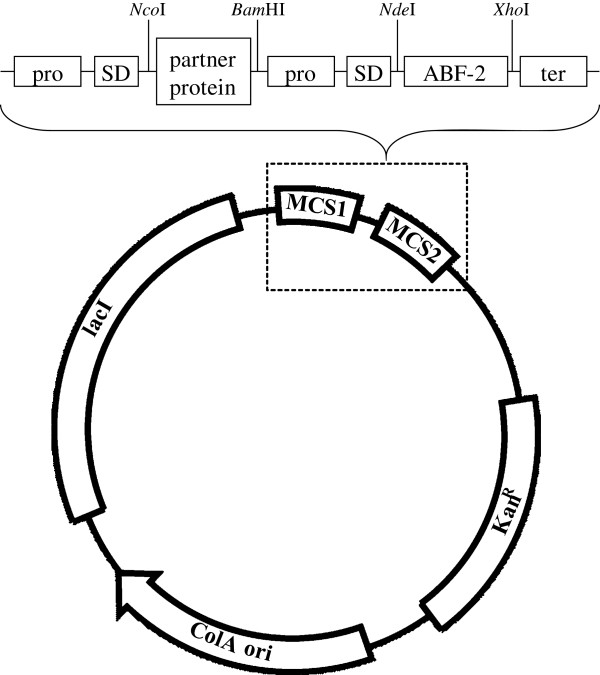
**Schematic representation of the expression vector.** pro, T7 promoter; SD, Shine-Dalgarno sequence; ter, T7 terminator.

The ABF-2 gene [GenBank: NM_058851] fragment was amplified by PCR with a set of primers using a cDNA-containing vector (Kato et al. 2002) as template (Table 1). This product was ligated to the pCOLADuet1 vector (Novagen) by using *Nde*І-*Xho*І sites (Figure 1), and the resulting vector was designated pCOLA-ABF-2. In this study, we selected four kinds of aggregation-prone proteins (human α-lactalbumin (HLA) [GenBank: NM_002289], bovine α-lactalbumin (BLA) [GenBank: NM_174378], human lysozyme (HLZ) [GenBank: NM_000239], and bovine lysozyme (BLZ) [GenBank: NM_180999]) as partner proteins. Each partner protein gene fragment was amplified by PCR with a set of primers using the cDNA-containing vector (Aizawa et al. 1998; Masaki et al. 2000; Nonaka et al. 2009) as template (Table 1). The PCR-amplified partner protein gene fragments (HLA, BLA, HLZ, and BLZ) were digested using restriction enzymes, and each was subcloned into the pCOLA-ABF-2 vector by using *Nco*І-*Bam*HІ sites. The gene fragments were included in the vector name; for example the pCOLA-ABF-2 vector containing the HLA gene was designated pCOLA-[HLA]-ABF-2. The clone sequence was confirmed by capillary sequencing.

**Table 1:** Sequences of primers used in this study

**Name**	**Primer sequence**^**a **^**(from 5′ end to 3' end)**	**Restriction site**
Primers for ABF-2 gene	F = GGAATTCCATATGGACATCG	*Nde*І
	ACTTTAGTACTTGTGC	
	R = CCGCTCGAGTTATCCTCTCT	*Xho*І
	TAATAAGAGCACCAAG	
Primers for HLA gene	F = GAATTCTCATGAAGCAATTC	*Bsp*HІ
	ACAAAATGTGAGCTG	
	R = CGGGATCCTTACAACTTCTC	*Bam*HІ
	ACAAAGCCACTG	
Primers for BLA gene	F = GAATTCCCATGGAACAGTTA	*Nco*І
	ACAAAATGTGAGGTG	
	R = CGGGATCCTTACAACTTCTC	*Bam*HІ
	ACAGAGCCA	
Primers for HLZ gene	F = GAATTCTCATGAAGGTCTTT	*Bsp*HІ
	GAAAGGTGTGAGTTG	
	R = CGGGATCCTTACACTCCACA	*Bam*HІ
	ACCTTGAACATAC	
Primers for BLZ gene	F = GAATTCTCATGAAGGTCTTT	*Bsp*HІ
	GAGAGATGTGAGC	
	R = CGGGATCCTTACAGGGTGCA	*Bam*HІ
	ACCCTCAA	

a. Restriction sites are underlined.

### Evaluating the effect of the partner protein on the ABF-2 expression level

*E. coli* BL21(DE3) cells were transformed with the various expression constructs (pCOLA-[HLA]-ABF-2, pCOLA-[BLA]-ABF-2, pCOLA-[HLZ]-ABF-2, pCOLA-[BLZ]-ABF-2, and pCOLA-ABF-2). The transformed cells were grown at 37°C in 5 mL of LB medium until the OD_600_ reached 1.0-1.2. The cells were induced by the addition of 1 mM isopropyl-β-D-thiogalactopyranoside (IPTG), cultivated for an additional 4 h, then harvested by centrifugation at 15,000 rpm for 5 min at 4°C. After the cells were lysed using Bugbuster (Novagen), inclusion bodies were isolated by centrifugation at 15,000 rpm for 5 min at 4°C and analyzed on Tricine-SDS PAGE. The intensity of ABF-2 bands was quantified by densitometry.

### Expression and purification of ABF-2

The *E. coli* strain BL21(DE3) harboring the pCOLA-[BLA]-ABF-2 vector was cultured overnight at 37°C in 50 mL of LB medium containing 20 μg/mL kanamycin. This preculture was inoculated in 1 L of medium (LB or ^15^ N-CHL) containing 20 μg/mL kanamycin. The culture was grown at 37°C, and protein expression was induced by the addition of 1 mM IPTG when the OD_600_ reached 1.0-1.2. At this point, for the production of ^15^ N-labeled ABF-2, a ^15^ N-labeled algal amino acid mixture was added to the CHL medium according to the protocol provided by the supplier. After an additional 4 h of cultivation, cells were harvested by centrifugation at 6,000 rpm for 10 min. The cells were resuspended in lysis buffer (20 mM Tris–HCl, 1 mM EDTA, pH 8.0) and disrupted by sonication. Next, inclusion bodies composed mainly of BLA and ABF-2 were isolated by centrifugation at 7,500 rpm for 30 min at 4°C. The inclusion bodies were washed twice with lysis buffer containing 0.1% TritonX-100 and washed once with lysis buffer (without TritonX-100).

The washed inclusion bodies were solubilized in solubilization buffer (8 M urea, 200 mM β-mercaptoethanol, 20 mM Tris–HCl, 3 mM EDTA, pH 8.0). After centrifugation at 7,500 rpm at 4°C for 30 min, the clarified supernatant was loaded onto a HiTrap SP HP cation-exchange column (GE Healthcare) pre-equilibrated with equilibration buffer (8 M urea, 20 mM Tris–HCl, 20 mM β-mercaptoethanol, 3 mM EDTA, pH 8.0). The bound ABF-2 was eluted with a linear gradient of equilibration buffer with 0–600 mM NaCl. The fractions containing ABF-2 were identified using Tricine-SDS PAGE. These fractions were collected and dialyzed three times against refolding buffer (20 mM Tris–HCl, 2 mM reduced glutathione, 0.2 mM oxidized glutathione, pH 8.0) for 12 h at 4°C. Correctly folded ABF-2 was purified by RP-HPLC on a Cosmosil 5C18-AR-300 column (Nacalai Tesque). The elution was carried out with a linear gradient of 25-45% acetonitrile with 0.1% trifluoroacetic acid. The yield of ABF-2 was determined by measuring the absorbance at 280 nm. The purified recombinant ABF-2 was lyophilized and stored at −30°C.

### Microbicidal assay

The antimicrobial activity of ABF-2 against *S. aureus* ATCC6538P and *E. coli* K12 was tested by a colony-forming unit assay. Bacteria were grown in tryptic soy broth (TSB) and collected in the mid-log phase by centrifugation. The bacteria were washed and diluted in sterile water. Various concentrations of ABF-2 were incubated with 1 × 10^7^ bacteria in a final volume of 50 μL at 37°C for 2 h. After incubation, 1,000-fold dilutions were prepared and 100 μL of the diluted samples was plated on a solid medium comprised of TSB. The plates were incubated for 20 h at 37°C and then the colonies were counted. The results indicated a 50% bactericidal concentration (BC_50_).

### Circular dichroism spectroscopy analysis

Circular dichroism (CD) spectra were measured in 1 mm pathlength quartz cuvettes on a J-725 spectropolarimeter (Jasco) equipped with a temperature control device. Spectra were acquired over a wavelength range between 250 nm and 200 nm as an average of four spectra with a 50 nm/min scan speed and a step resolution of 0.1 nm at 25°C. ABF-2 samples (50 μM) were used in buffer (20 mM Tris–HCl, pH 8.0). Buffer blanks were subtracted from all spectra. All measurements were averaged and converted to molar ellipticity.

### NMR spectroscopy

^15^ N-labeled ABF-2 was dissolved in 20 mM phosphate buffer (pH 5.7) comprising a 90% H_2_O/10% D_2_O mixture. NMR experiments were performed on a JEOL ECA-600 spectrometer and a Bruker AVANCE ІІІ 800 spectrometer with a TCI cryogenic probe. The ^1^H-^15^N HSQC spectrum was collected at 20°C. The data were processed by using NMRpipe/NMRdraw software (Delaglio et al. 1995).

## Results

### Construction and expression of coexpression plasmids encoding ABF-2 and partner proteins

**Figure 2:**
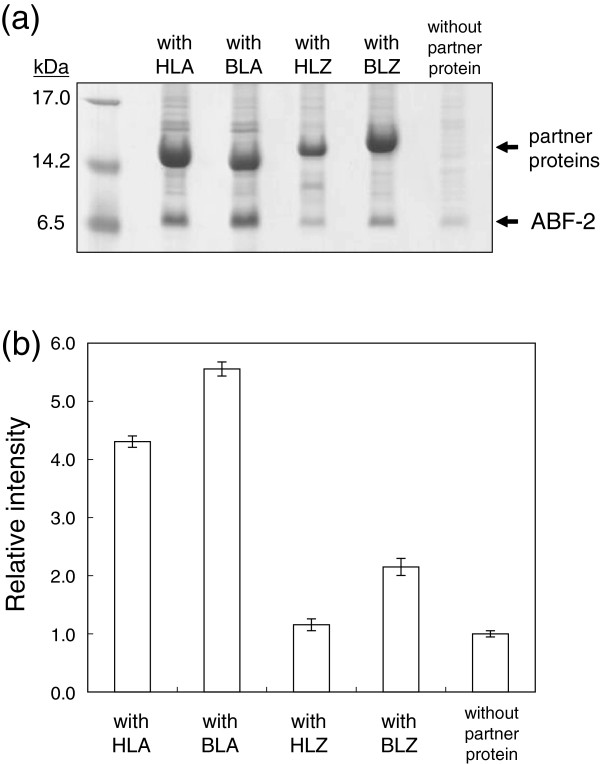
**Effects of the charges of partner proteins on the ABF-2 expression level. (a)** Tricine-SDS-PAGE analysis of the expression level of ABF-2. **(b)** The intensity data of the coexpression method are expressed in relation to those for the direct expression method. The graph represents the average intensities of three independent experiments.

First, we tried to express ABF-2 as an inclusion body in *E. coli*, but the expression level of ABF-2 was extremely low (Figure 2). To enhance the inclusion body formation of ABF-2, we decided to apply a coexpression method.

To construct a coexpression plasmid containing ABF-2 and aggregation-prone partner protein genes, we utilized the commercially available pCOLADuet1 vector from Novagen. This vector has two RBS sites flanking two multiple cloning sites, which are under the control of their respective T7 promoters. In this study, partner protein genes were subcloned into the first multiple cloning site of the pCOLA vector, and the ABF-2 gene was subcloned into the second multiple cloning site of the pCOLA vector.

To evaluate the effect of a partner protein on the ABF-2 expression level, various partner proteins and ABF-2 were coexpressed (Table 2). The expression of ABF-2 was moderately increased in the case of the coexpression of ABF-2 and BLZ (Figure 2). The expression of ABF-2 as an inclusion body was markedly increased by coexpression of HLA or BLA. On the other hand, the expression level of ABF-2 was not affected, although HLZ was clearly overexpressed as an inclusion body. Because the expression level of ABF-2 was increased the most by coexpression of BLA, we selected BLA as a partner protein for the large-scale production of ABF-2.

**Table 2:** Properties of antibacterial factors and partner proteins used in this study

**Name**	**Mw**	**pI**	**GRAVY score**^**a**^
Antibacterial factor 2 (ABF-2)	6999.2	9.1	−0.072
Human α-lactalbumin (HLA)	14078.2	4.7	−0.255
Bovine α-lactalbumin (BLA)	14186.1	4.8	−0.453
Human lysozyme (HLZ)	14700.7	9.3	−0.485
Bovine lysozyme (BLZ)	14415.2	6.5	−0.395

a. GRAVY stands for grand average of hydropathy. The Positive GRAVY scores indicate hydrophobic peptides, and negative scores indicate hydrophilic peptides.

### Purification and refolding of ABF-2

**Figure 3:**
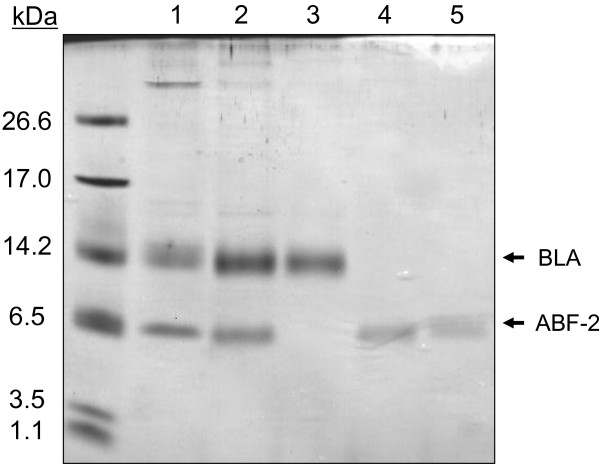
**Expression and purification of recombinant ABF-2.** Lane 1: Inclusion body after ultrasonication and centrifugation. Lane 2: Solubilized inclusion body. Lane 3: A flowthrough fraction that was passed through cation-exchange chromatography. Lane 4: Purified ABF-2 using cation-exchange chromatography. Lane 5: Purified correctly folded ABF-2 using RP-HPLC.

ABF-2 was easily separated from BLA by using cation-exchange chromatography under denaturing conditions, because of the opposite charge (Figure 3). Unlike in fusion protein systems, there was no need to remove the fusion protein tag by enzymatic or chemical methods. After cation-exchange chromatography, we obtained about 54 mg of crude ABF-2 without disulfide bonds from 1 L of *E. coli* culture. Next, this crude ABF-2 was refolded by dialysis. After the refolding and purification procedure, we obtained 7.8 mg of correctly folded ABF-2 from 1 L of culture.

### Antimicrobial activity of recombinant ABF-2

The microbicidal activity of recombinant ABF-2 was examined (Table 3). The 50% microbicidal concentrations were 0.01 and 0.1 μM for *S. aureus* and *E. coli*, respectively. The Gram-positive bacterium (*S. aureus*) was 10-fold more sensitive than the Gram-negative bacterium (*E. coli*). Neither *S. aureus* nor *E. coli* was sensitive to the misfolded fraction (data not shown).

**Table 3:** Microbicidal activity of recombinant ABF-2

**Organism**	**BC**_**50 **_**(μM)**
Gram-positive bacteria	0.01
*Staphylococcus aureus* (ATCC6538P)	
Gram-negative bacteria	0.1
*E. coli* (K12)	

Microbicidal activity was assessed as the 50% microbicidal concentration (BC_50_).

### Circular dichroism spectrum of ABF-2

**Figure 4:**
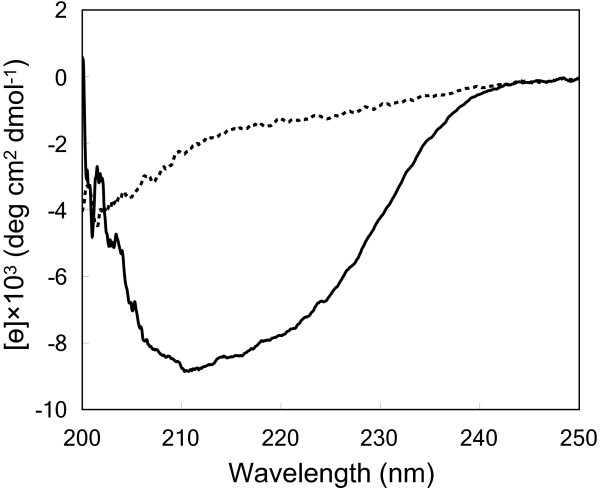
Circular dichroism spectra of correctly folded (solid line) and misfolded (dotted line) ABF-2.

Furthermore, to confirm that purified ABF-2 was folded correctly, the CD spectra of ABF-2 were measured. The spectra of misfolded ABF-2 revealed a random coil structure (Figure 4). On the other hand, the CD spectra of refolded ABF-2 showed a significant negative band in the region between 208 and 220 nm. This indicates that a secondary structure was formed in refolded ABF-2.

### ^1^H-^15^N HSQC spectrum of ^15^ N-labeled ABF-2

**Figure 5:**
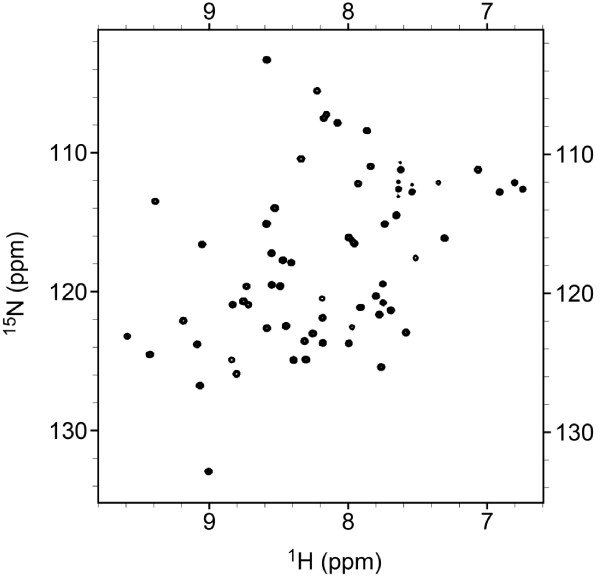
^**1**^**H-**^**15**^**N HSQC spectrum of 0.7 mM **^**15**^ **N-labeled ABF-2.**

The large amount of isotopically labeled ABF-2 enabled the rapid and sensitive acquisition of NMR spectra. The HSQC spectrum of ^15^ N-labeled ABF-2 is presented in Figure 5. The number of peaks in the HSQC spectrum of ^15^ N-labeled ABF-2 corresponded approximately to the number of residues in ABF-2. The majority of the ^1^H-^15^N cross-peaks lay between 7.5 and 9.5 ppm. The sharp, well-dispersed peaks indicate that purified ABF-2 was correctly folded.

## Discussion

Because the correct disulfide arrangements of disulfide-containing peptides are difficult to obtain, the yeast *P. pastris* is widely used to express peptides containing intramolecular disulfide bridges. The main advantage of *P. pastris* as a host is that it is expected to secrete peptides with correct disulfide bridges directly into culture medium (Daly and Hearn 2005). Several CSαβ-type antimicrobial peptides have been produced by using *P. pastris*, and these were well characterized (Wang et al. 2008; Zhang et al. 2011; Wiens et al. 2011). However, a previous study showed that *P. pastris* is not a suitable host for large-scale production of ABF-2. Although the antimicrobial spectrum of ABF-2 was investigated, structural and mutational studies of ABF-2 have not yet been conducted because of the low yield of ABF-2 in *P. pastris* (Kato et al. 2002). Therefore, it is very important to develop an alternative expression method that enhances the expression level of ABF-2.

In this study, we selected *E. coli* as an expression host and at first tried to express ABF-2 directly in *E. coli* as an inclusion body. Unfortunately, the direct expression of ABF-2 in *E. coli* was insufficient due to the instability of the expressed ABF-2 itself in the cells (Figure 2). We speculated that this low-level expression of ABF-2 was caused by the degradation of expressed ABF-2 by proteases of *E. coli*. Therefore, we considered that the prevention of ABF-2 degradation by proteases is essential to enhance the expression level of ABF-2 as an inclusion body.

To prevent the degradation of target peptides, fusion protein systems have been used in many studies. The attachment of soluble proteins to target peptides has been observed to prevent degradation and promote proper folding (Xu et al. 2006). However, when an antimicrobial peptide such as ABF-2 is expressed in a soluble form, it may damage host cells by disrupting their cell membranes. Moreover, in fusion protein systems, enzymatic or chemical cleavage is inevitable to remove the fusion protein tag. Enzymatic cleavage often degrades recombinant peptides, because widely used proteases, such as enterokinase and factor Xa, often show nonspecific cleavages at unexpected sites. Furthermore, many peptides contain potential cleavage sites cleaved by chemicals. For instance, CNBr is commonly used to cleave peptide bonds C-terminal to methionine residues in proteins and peptides. However, because ABF-2 contains methionine in its amino acid sequence, CNBr cannot be used to separate ABF-2 from the fusion protein.

Expression of inclusion bodies may also be useful to avoid proteolytic degradation of the target peptide. Walsh et al. succeeded in expressing a large amount of amyloid β-peptide (Aβ) directly as an inclusion body (Walsh et al. 2009). Because the Aβ peptide has a strong intrinsic propensity to aggregate, we considered that overexpression of the Aβ peptide would readily lead to the formation of an inclusion body in *E. coli*. This method is a very simple and inexpensive way to produce a large amount of Aβ peptides. However, it is generally difficult to control the inclusion body formation in *E. coli* cells, especially in the case of small and common peptides. As another way to produce fusion proteins, the utilization of insoluble protein tags has also been reported (Park et al. 2009). This method is expected to prevent both degradation and toxicity. However, when an insoluble protein tag is used for the fusion expression, it is difficult to employ enzymatic cleavage due to the insolubility of the fusion protein, and chemical cleavage is usually necessary. This cleavage step is thus one of the drawbacks of this method.

In some studies, the coexpression of an insoluble partner protein has been reported to enhance the inclusion body formation of the target peptide and protect it from proteolytic degradation by protease. Saito *et al*. reported that the expression level of somatomedin C was enhanced in the case of coexpression of insulin-like growth factor І (Saito et al. 1987). Jang *et al*. succeeded in producing a potent antimicrobial peptide, buforin ІІb, by coexpression of human gamma interferon (Jang et al. 2009). To obtain a large amount of ABF-2, we decided to apply this method to ABF-2 and examine the effects of aggregation-prone partner proteins in detail.

In previous studies, translationally coupled two-cistron plasmids were used to coexpress target peptides and partner proteins (Saito et al. 1987; Jang et al. 2009). However, genetic manipulation was needed to construct the translationally coupled two-cistron expression systems. In the present study, we simply used the commercially available pCOLADuet-1 vector to coexpress ABF-2 and partner protein genes (Figure 1). Because this vector is designed for the cloning and coexpression of two genes, the construction of a coexpression plasmid is very easy. Therefore, our method can be easily applied to many proteins and peptides.

In this study, we chose four kinds of proteins as aggregation-prone partners. Lysozyme and α-lactalbumin appear to have evolved from a common ancestral protein, as evidenced by the similarity of their amino acid sequences and three-dimensional structures as well as by the high conservation of disulfide bridges (McKenzie and White 1991). Some studies have clearly shown that these four proteins each form a large amount of inclusion bodies when overexpressed in *E. coli* (Svensson et al. 2000; Li and Su 2002). Interestingly, the isoelectric points of these proteins are different despite their sequential similarity. Thus, we selected these proteins as good models of aggregation-prone partners in order to evaluate the effect of the charge of the partner protein on the expression level of cationic ABF-2 (pI 9.1).

Coexpression of BLZ, whose isoelectric point is 6.5, modestly enhanced the expression level of ABF-2. In contrast, ABF-2 was produced effectively as an inclusion body in the case of coexpression with an anionic partner protein (HLA or BLA). Coexpression of HLZ, whose isoelectric point is high, yielded no change in the expression level of ABF-2. Although it has been conjectured that the charge of both target and partner proteins influences inclusion body formation, the effect of the charge of the partner protein on the expression level of the target peptide has not been elucidated in detail. In this study, we experimentally showed that the charge of the partner protein is an important factor for enhancing the inclusion body formation of the target peptide. Interestingly, although HLA and BLA have almost the same isoelectric points and molecular weights, the expression level of ABF-2 was enhanced more by coexpression of BLA than by that of HLA. Because not only electrostatic but also hydrophobic interactions are thought to be critical factors for inclusion body formation (Murby et al. 1995), we compared their GRAVY scores, which express the total hydrophobicity of a protein. However, we could not find a correlation between the expression level of ABF-2 and the GRAVY scores of the partner proteins. There may be unknown factors that affect inclusion body formation.

It has been reported that the presence of impurities in an inclusion body, such as nucleic acids and non-plasmid-encoded proteins, affects the final refolding yield of the target (Maachupalli-Reddy et al. 1997). These impurities can be removed by washing the body using a low concentration of detergent. Therefore, inclusion body washing is very important to enhance the refolding yield of the target. However, in this study we observed that the inclusion body composed of ABF-2 without partner proteins was gradually solubilized during the washing process (data not shown). Because of this unfavorable solubilization of the body, ABF-2 was lost during the washing process and we could not obtain even crude ABF-2 in the experiment using expression without partner proteins. On the other hand, the inclusion body composed of ABF-2 and the partner protein showed almost no solubilization during the washing process, suggesting that a robust inclusion body was formed via coexpression of the peptide and a partner protein. From these results, it can be said that coexpression of an aggregation-prone protein is effective not only to enhance the expression level of a target peptide as an inclusion body but also to prevent the unfavorable loss of an inclusion body during washing.

Because the charge of ABF-2 is opposite that of BLA, we succeeded in separating ABF-2 from BLA efficiently by a simple one-step cation-exchange chromatography without enzymatic or chemical cleavage (Figure 3). After cation-exchange chromatography, we succeeded in obtaining 54 mg of crude ABF-2 without disulfide bridges from 1 L of culture. Although crude ABF-2 was refolded by a very simple standard dialysis refolding protocol, we obtained as much as 7.8 mg of correctly folded ABF-2. Because refolding additives, such as arginine, have been used to suppress the aggregation of proteins during refolding in many studies (Arakawa et al. 2007), optimization of the refolding protocol may enhance the refolding yield of ABF-2.

To confirm that refolded ABF-2 was correctly folded and active, purified ABF-2 was evaluated by a colony-forming unit assay as well as by CD and NMR spectroscopy (Table 3, Figures 4, 5). We confirmed that purified ABF-2 was active against both *S. aureus* and *E. coli*. Moreover, *S. aureus* is more sensitive than *E. coli*. These results were identical to the findings reported previously (Kato et al. 2002). Quantitative analysis of the CD spectra of purified ABF-2 indicated a secondary structural content that included 28% α-helix and 17% β-sheet. This result was in agreement with the categorization of ABF-2 as a CSαβ-type antimicrobial peptide. Moreover, the HSQC spectrum of ^15^ N-labeled ABF-2 is sharp and well dispersed. From these data, we concluded that purified ABF-2 was correctly folded. This sample will enable us to analyze the structure and molecular mechanism of ABF-2 by using NMR in future studies.

It is known that the long C-terminal regions of ABFs are divergent and vary in length (Froy 2005). Therefore, the difference in the C-terminal region of ABFs is thought to affect their antimicrobial spectrum. We believe that the method described in this work could be applied not only to ABF-2 but also to other ABFs. In future studies, we plan to use this technique to elucidate the effects of the differences in the C-terminal regions of ABFs on their antimicrobial spectra.

Here, we demonstrated that ABF-2 could be expressed as an inclusion body in large quantities by coexpression of an aggregation-prone protein. Moreover, the expression level of ABF-2 was greatly enhanced by coexpression of anionic partner proteins. After the refolding and purification processes, we obtained milligram quantities of correctly folded ABF-2. Finally, this expression method allowed stable isotopic labeling of recombinant ABF-2, which is required for structural studies using multidimensional heteronuclear NMR spectroscopy.

## Competing interests

The authors declare that they have no competing interests.
